# Antibiofilm approaches as a new paradigm for treating infections

**DOI:** 10.1088/2516-1091/ad1cd6

**Published:** 2024-02-09

**Authors:** Fany Reffuveille, Yasser Dghoughi, Marius Colin, Marcelo D T Torres, Cesar de la Fuente-Nunez

**Affiliations:** 1 Université de Reims Champagne-Ardenne, Biomatériaux et Inflammation en Site Osseux, BIOS EA 4691, SFR Cap Santé, 51097 Reims, France; 2 Université de Reims Champagne-Ardenne, UFR Pharmacie, Service de Microbiologie, 51097 Reims, France; 3 Machine Biology Group, Departments of Psychiatry and Microbiology, Institute for Biomedical Informatics, Institute for Translational Medicine and Therapeutics, Perelman School of Medicine, University of Pennsylvania, Philadelphia, PA, United States of America; 4 Departments of Bioengineering and Chemical and Biomolecular Engineering, School of Engineering and Applied Science, University of Pennsylvania, Philadelphia, PA, United States of America; 5 Department of Chemistry, School of Arts and Sciences, University of Pennsylvania, Philadelphia, PA, United States of America; 6 Penn Institute for Computational Science, University of Pennsylvania, Philadelphia, PA, United States of America

**Keywords:** antibiofilm strategies, antibiofilm molecules, biofilm models, engineering approaches, computational approaches

## Abstract

The lack of effective antibiotics for drug-resistant infections has led the World Health Organization to declare antibiotic resistance a global priority. Most bacterial infections are caused by microbes growing in structured communities called biofilms. Bacteria growing in biofilms are less susceptible to antibiotics than their planktonic counterparts. Despite their significant clinical implications, bacterial biofilms have not received the attention they warrant, with no approved antibiotics specifically designed for their eradication. In this paper, we aim to shed light on recent advancements in antibiofilm strategies that offer compelling alternatives to traditional antibiotics. Additionally, we will briefly explore the potential synergy between computational approaches, including the emerging field of artificial intelligence, and the accelerated design and discovery of novel antibiofilm molecules in the years ahead.

## 1. Introduction

Biofilms are diverse communities of bacteria, consisting of various cell types such as active or dormant cells and persisters, enclosed within an exopolymeric matrix. This intricate structure provides bacteria with the ability to withstand the effects of antimicrobial agents (de la Fuente-Núñez *et al* 2013). Scientists have been exploring different approaches to combat biofilms, including prevention and eradication.

Prevention involves the introduction of molecules before biofilm formation to impede its development. On the other hand, eradication focuses on targeting mature biofilms that have already formed prior to the application of treatment.

While numerous molecules have demonstrated the ability to eradicate biofilms in laboratory settings (*in vitro* models), their effectiveness in real-life scenarios (*in vivo* models) has been limited (de la Fuente-Núñez *et al* 2013, Agbale *et al* 2019, Silveira *et al* 2021). This discrepancy can be attributed to various environmental factors influencing biofilm formation. These factors include the adhesion capabilities of the constituent bacteria to living or non-living surfaces, as well as the availability of oxygen and nutrients, all of which significantly impact the structure and composition of biofilms (Arciola *et al* 2018, Reffuveille *et al* 2018, Oliveira *et al* 2018, Crabbé *et al* 2019, Bidossi *et al* 2020, Lamret *et al* 2021).

Given the current understanding of biofilms, it is imperative to urgently develop new antibiofilm strategies that account for these recent insights and challenges associated with biofilm control.

### 1.1. Biological approaches

In the race against the emergence of antibiotic resistance, finding new antibiotics has become a top priority. Equally important are the development of new therapies (*e.g.,* bacteriophages, antimicrobial peptides (AMPs), or probiotics) and the repurposing of old molecules and previously abandoned antibiotics. Biofilms are associated with two-thirds of all infections in humans, yet conventional antibiotics have been selected or designed to target planktonic bacteria and are usually not effective against biofilms. The antibiofilm activity of molecules has not been a focus of massive screening efforts, and antibiofilm compounds have not yet been approved for human use. As conventional antibiotic discovery platforms currently overlook the majority of infections, it is clear that these platforms should be expanded to include the investigation of antibiofilm properties.

## 2. Antibiotics and antibiofilm activity

The usual targets of recently developed antibiotics are cell wall synthesis, cell membranes, and DNA or protein synthesis, in short, sites that distinguish bacteria from their eukaryotic counterparts. Some of the molecules known to attack these targets have been shown to possess antibiofilm activity and are discussed next.

### 2.1. Cell wall targets

Among the new cephalosporins that target cell wall synthesis, ceftarolin and ceftopibrole have been identified as excellent inhibitors of biofilms formed by methicillin-resistant *Staphylococcus aureus* (MRSA) in a cystic fibrosis lung biofilm model (Boudet *et al* 2021). Ceftarolin, which inhibits cell wall synthesis by blocking extracytoplasmic transpeptidase, is effective at preventing biofilm formation not only by MRSA (Lázaro-Díez *et al* 2016) but also by Gram-positive bacteria more generally (Thieme *et al* 2018), including methicillin-resistant *Staphylococcus epidermidis* (Domínguez-Herrera *et al* 2016). However, ceftarolin is unable to eradicate mature biofilms formed by Gram-positive bacteria and, when combined with ampicillin, actually enhances the formation of small colony variants which compose biofilm and are tolerant to antimicrobial treatment (Thieme *et al* 2018). Additionally, the combination of ceftarolin with daptomycin enhances biofilm formation (Barber *et al* 2015). Ceftobiprole, a more promising cephalosporin, is active alone or in combination with rifampicin against staphylococci in a Minimal Biofilm Eradication Concentration (MBEC) model at dilutions of the maximum free-drug plasma concentration (*fc*_max_) attained during clinical use (*fc*_max_; superior to 30 mg l^−1^) until 0.25 × *fc*_max_ (Abbanat *et al* 2014). Ceftobiprole has thus become a good candidate for treating infections associated with foreign bodies with a MIC always under 2 mg l^−1^ (Hischebeth *et al* 2018).

One promising drug that has shown an antibiofilm effect on Gram-negative bacteria is cefiderocol, a bacteriostatic cephalosporin that also displays siderophore activity. Cefiderocol effectively reduces biofilm formation (by 90% at a concentration of 4 *μ*g ml^−1^) and potently inhibits the planktonic growth of a range of medically important Gram-negative pathogens (Pybus *et al* 2021). In particular, cefiderocol is efficient at preventing the formation of *Klebsiella pneumoniae* (Bao *et al* 2021) and *Acinetobacter baumannii* biofilms (Cipko *et al* 2021).

Lipoglycopeptides are another class of antibiotics that arrest peptidoglycan synthesis. In particular, the lipoglycopeptide dalbavancin eradicates biofilms formed by *S. aureus* (MBEC_90_ = 2 *μ*g ml^−1^)*, S. epidermidis* (MBEC_90_ = 4 *μ*g ml^−1^), and enterococci (MBECs for vancomycin-susceptible and -resistant isolates were ⩽4 and >16 *μ*g ml^−1^) but is inefficient against vancomycin-resistant strains biofilms (Fernández *et al* 2016, Neudorfer *et al* 2018, Silva *et al* 2021). As catheters offer optimal conditions for bacterial adhesion, they are often at the origin of biofilm formation (Costerton *et al* 1999). To prevent it, a catheter lock solution was developed by combining dalbavancin with heparin; this lock fills the catheter when it is not in use, preventing bacterial attachment to the catheter wall and subsequent biofilm formation (Díaz-Ruíz *et al* 2018). Moreover, an additive effect against *S. aureus* was observed *in vitro* when dalbavancin was used in combination with rifampicin (Jacob *et al* 2021), but rifampicin resistance emerged *in vivo* (El Haj *et al* 2022). The combination of dalbavancin with biofilm-detaching compounds needs to be carefully studied as it may vary depending on the combinations used. For example, the molecule *N*-acetylcysteine (NAC) is known to perturb biofilm structure; however, antagonism has been observed between NAC and dalbavancin. On the other hand, ficin, a protease known for cleaving biofilm matrix proteins, displays an additive effect when combined with dalbavancin on staphylococcal biofilms (Žiemytė *et al* 2020).

The bacterial outer membrane, a component of the cell wall of Gram-negative bacteria, is also a promising antimicrobial target. Ceragenins or cationic steroid antimicrobials (CSAs) are molecules having a sterol backbone with amino acids and other chemical compounds. Their positive charge and amphipathic nature allow their insertion in the membrane and its subsequent disruption. Another property of CSAs is their ability to bind lipid A from lipopolysaccharides, leading to an anti-inflammatory effect. Moreover, ceragenins potently prevent *Pseudomonas aeruginosa* biofilm formation at 50 *μ*g ml^−1^ (Paprocka *et al* 2021).

### 2.2. Cytoplasmic targets

Antibiotics can also target cytoplasmic components of bacteria, most commonly, DNA synthesis and protein synthesis. Delafloxacin, a fluoroquinolone that inhibits DNA topoisomerase IV activity, penetrates the biofilm matrix more effectively than other conventional antibiotics such as vancomycin and daptomycin (Siala *et al* 2014). Eravacyclin is a fluorocycline that binds the bacterial ribosome, blocking protein synthesis. Although eravacyclin has poor activity in eradicating staphylococcal biofilms (Zhuchenko *et al* 2020), it can destroy biofilms formed by uropathogenic *Escherichia coli* at 0.5 *μ*g ml^−1^ (Grossman *et al* 2015). Tedizolide, another molecule that blocks ribosomes, is used for the treatment of skin and soft tissue infections as it inhibits biofilm formation by staphylococci at concentrations lower than its MIC for planktonic bacteria, but it is inactive *in vitro* against mature *S. aureus* biofilms (Abad *et al* 2019). Furthermore, Bayer *et al* (2016) observed efficient activity of tedizolide against established *S. aureus* biofilm in a murine model. In association with rifampicin, tedizolide reduced biofilm biomass, and rifampicin-resistant strains were not identified after antibiotic exposure, thus validating this approach (Gidari *et al* 2020).

The development of antibiotics often involves testing their interactions, as synergy is known to reduce the likelihood that bacterial resistance will develop. In a recent study, monotherapy with ceftolozane (cephalosporin) or tazobactam had low bactericidal activity, but when ceftolozane was combined with colistin, the cocktail efficiently treated multidrug-resistant *P. aeruginosa* biofilm infection (Gómez-Junyent *et al* 2019). Certain antibiotic combinations have been shown to be effective only against planktonic cells, whereas others have shown more promise at targeting biofilms. For example, the combination of ceftazidime/avibactam plus meropenem had synergistic antibacterial activity against planktonic *K. pneumoniae* but displayed weak antibiofilm activity, suggesting that desirable clinical outcomes for treating infections caused by biofilms may be more difficult to achieve, even with a combination of synergistic antibiotics (Papalini *et al* 2020).

Older and underused antibiotics, including last-resort antibiotics, such as colistin, can be combined with other antibiotics. Colistin is a polymyxin antibiotic (i.e. a polypeptide); its combination with fosfomycin showed synergy against multidrug-resistant Gram-negative pathogens grown in biofilms and reduced the emergence of resistance to both antibiotics (Boncompagni *et al* 2022).

## 3. New therapies

Aside from antibiotics, new therapies such as bacteriophage cocktails, are also emerging. Recent studies, for example, have demonstrated the efficacy of bacteriophages against MRSA, especially in association with daptomycin and ceftaroline with a >3 log_10_ CFU ml^−1^ reduction (Kebriaei *et al* 2022), and against *P. aeruginosa* in difficult-to-treat bone and soft tissue infections in combination with low ceftazidime-avibactam concentrations (two-fold lower than the standalone needed concentrations) (Racenis *et al* 2022).

Probiotics represent another alternative and relatively new therapeutic approach against infections: for example, the use of beneficial lactic acid bacteria led to the disruption and the removal of staphylococcal biofilms.

*In vitro* studies have shown that lactic acid bacteria have the ability to remove and substitute pathogenic *S. aureus* biofilms with beneficial biofilms. However, implementing probiotic bacterial strains as a viable strategy can be challenging, as only two specific probiotic strains have demonstrated the capability to form a biofilm that can effectively replace the pathogenic bacteria. Achieving complete replacement of the *S. aureus* biofilm with lactic acid bacteria is crucial to ensure its thorough eradication (Wallis *et al* 2019).

AMPs constitute another class of promising antibiotic molecules and represent an alternative to conventional therapies. These molecules are produced in various organisms, including plants, insects, and mammals, and have been studied for their immunomodulatory properties and potent antimicrobial activity (Overhage *et al* 2008, de la Fuente-Núñez *et al* 2014, 2015a, 2015b). Inspired by nature, scientists have developed synthetic AMPs. Interestingly, some of the synthetic AMPs exhibit excellent broad-spectrum antibiofilm activity, and both prevent and eradicate biofilms formed by numerous Gram-positive and Gram-negative bacterial species at low concentrations (from 2.5 to 20 *μ*g ml^−1^) (de la Fuente-Núñez *et al* 2014). These peptides have also been studied for their ability to synergistically interact with different classes of antibiotics against biofilms (Reffuveille *et al* 2014). In addition, noncanonical amino acids have been incorporated into peptide sequences to confer protection to the peptide against proteolytic degradation (de la Fuente-Núñez *et al* 2015b, Cesaro *et al* 2022). The comparison between antibiofilm biological approaches described in this review (table 1) highlights that bacteriophages and AMPs are promising strategies to counter biofilm infections.

### 3.1. Biofilm models

Current models used in antibiofilm studies do not consider the whole environment of the infected site, including the presence of biotic (e.g., skin, mucous membrane, bone) or abiotic (e.g., prosthesis, catheter) supports, dioxygen, ions, and nutrient concentrations, or even the presence of immune and non-immune cells). Altogether, these factors could help explain the failure rate of antibiofilm compounds used thus far in *in vivo* models. New *in vitro* models are currently being developed to screen antibiofilm molecules more accurately. Traditionally, new antibiotic candidates have only been tested against planktonic organisms without considering biofilm-related resistance. Two major types of biofilm models, static and dynamic, have been developed to assess the antibiofilm potential of compounds.

**Figure 1.**
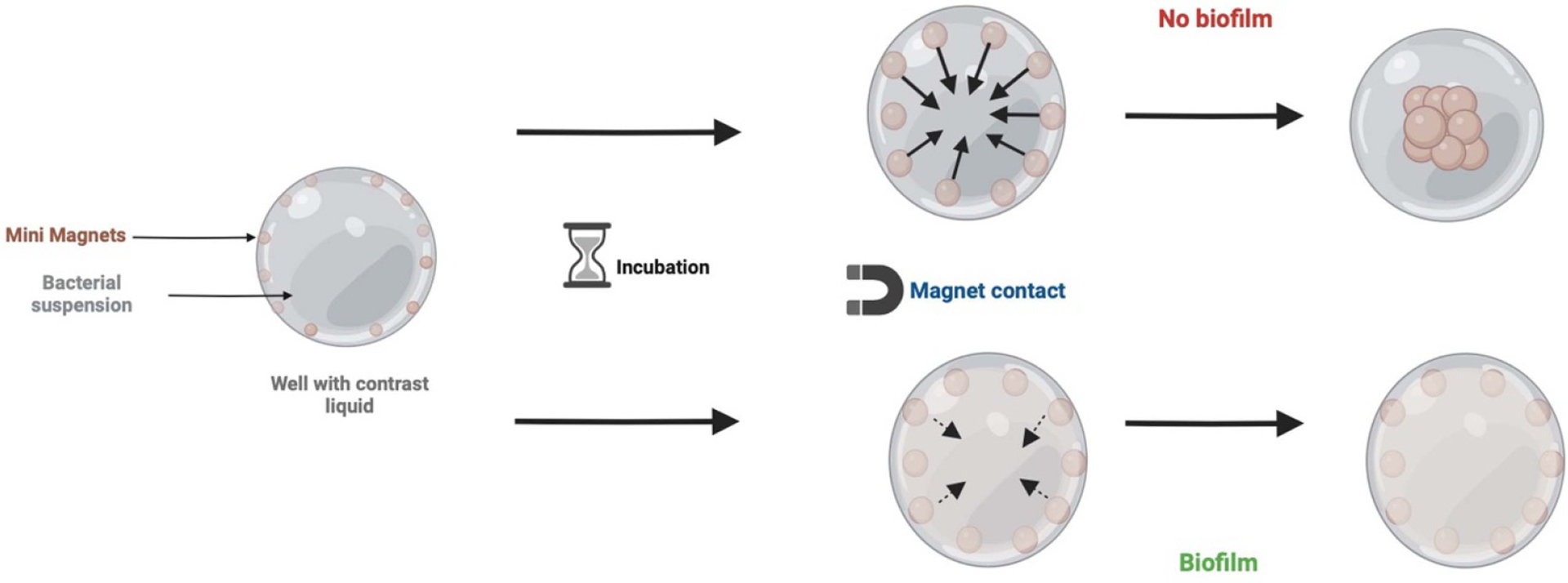
Principle of the Biofilm Ring Test (BRT). When a biofilm forms, the magnetic beads in the culture medium become immobilized or blocked, whereas in the absence of a biofilm, the beads remain freely mobile. By applying a magnetic force, it is possible to detect the presence of a biofilm if the beads do not aggregate or regroup. This scheme was created in BioRender.com.

Static models can be used to rapidly analyze the kinetics of biofilm formation. For example, the Biofilm Ring Test (BRT), widely used as a laboratory test, is an informative tool used to characterize biofilms (Olivares *et al* 2016) (figure 1). The principle underlying this method is that cells in biofilms, as well as being attached to each other, are anchored to a substrate, whereas planktonic cells are not. To perform the BRT, a bacterial suspension is mixed in a polystyrene microplate with 2–10000000 magnetic microbeads per well. The beads have to be magnetic or to contain magnetizable particles, and their dimension can range between 10 nm to 100 *μ*m. Initially, the beads are placed in the center of the well and are free to move. After incubation, the mobility of the microbeads is measured by using a magnet to draw the beads together. A magnetic, electric, or electromagnetic field generated by a magnet or solenoid is used with a magnetic force according to supplier information. If a biofilm is formed, the beads are immobilized, whereas if biofilm formation is weak, the beads can move. This technology can help to classify the bacterial strains into three groups: (1) strains that form a solid biofilm in less than 2 h; (2) strains that progressively set up a biofilm within 24 h; and (3) strains that stay in the planktonic phase. BRT can be used with a bacterial suspension with antibiotics to select which antibiotics inhibit biofilm formation, to define the time needed to observe this activity, and to evaluate whether single or repeated antibiotic administration is needed to treat infections caused by the bacterial strain of interest. Unlike microscopy techniques, which can require laborious sample preparation and has the associated risks of loss or detachment of the bacteria through staining techniques and washing steps, BRT appears to be an easy and innovative technique for routine biofilm applications (Olivares *et al* 2016).

**Figure 2.**
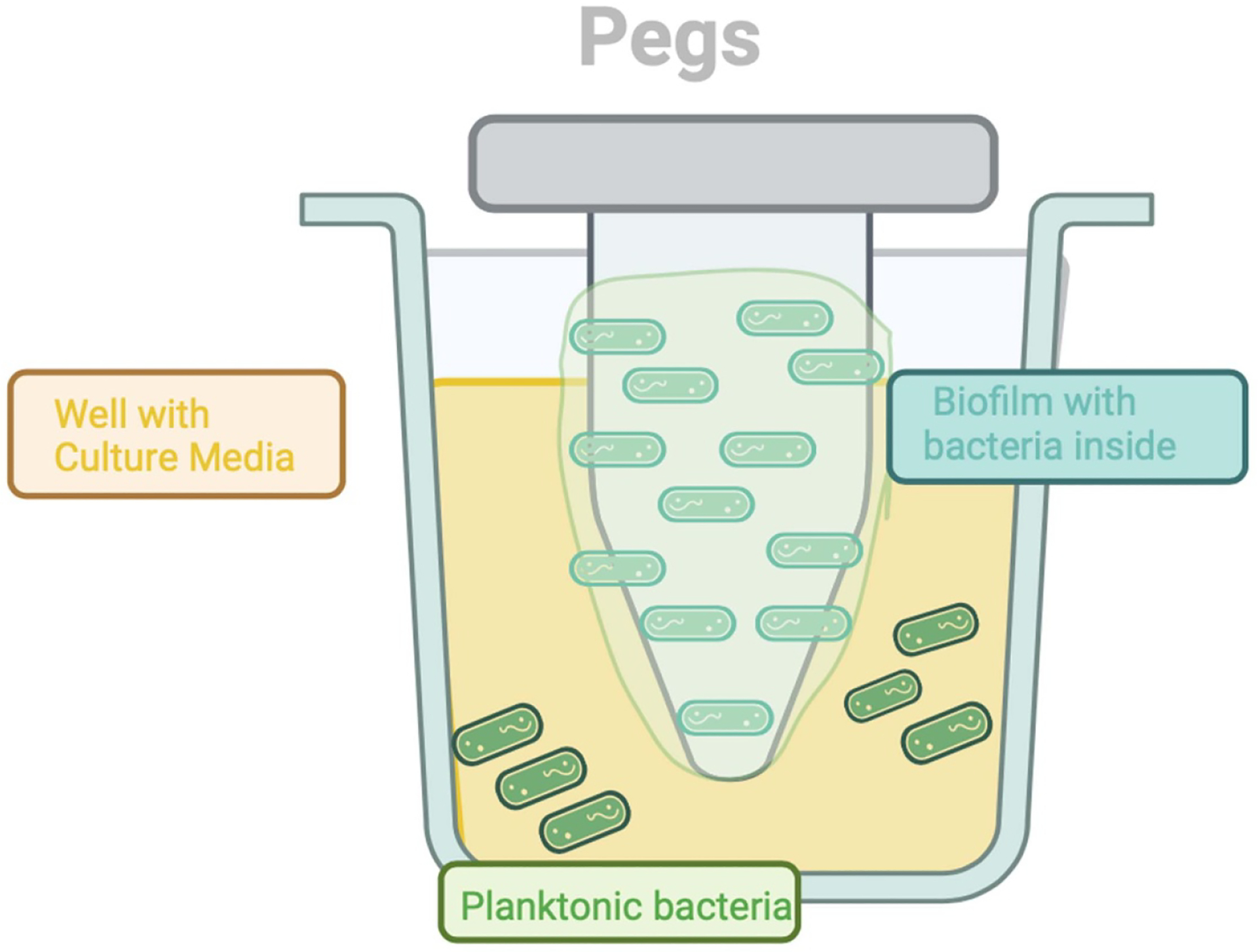
Mechanism of Calgary Biofilm Device (CBD), inspired from Ceri *et al* (1999). This scheme was created in BioRender.com.

The Calgary Biofilm Device (CBD), a static model, was initially developed in the early 2000s (Ceri *et al* 1999) as a new way to screen antibiotics and biocides for activity against microbial biofilms (figure 2). The implementation of CBD made it possible to rapidly evaluate the antibiofilm effects of a range of molecules (Ceri *et al* 1999). With this tool, the biofilm can be formed by the adherent bacteria on a peg attached to the lid of a tube or well, while the planktonic cells remain in the wells. After inoculating the bacteria at a defined concentration, plates are incubated for a specific time to obtain the biofilm; incubation times vary between 6 and 144 h, depending on the tested species or the study aims (Ceri *et al* 1999). Biofilms can be removed either from individual pegs, which can be broken off from the lid, or from all pegs at one time, by the application of 5 min of sonication in an ultrasonic bath. The CBD model makes it possible to test biofilm susceptibility to antimicrobial agents. The MBEC, defined as the minimal concentration of antibiotic required to eradicate the biofilm, is calculated by enumerating the surviving bacteria. CBD is the first system specifically adapted to antibiotic susceptibility testing for adherent bacterial populations. Moreover, this technology reduces the time required to evaluate the susceptibility of a bacterial strain to antibiotics and can be applied to recalcitrant, recurrent, or device-related infections caused by organisms for which MICs have not provided clinically relevant information (Ceri *et al* 1999).

New tools are currently being developed to rapidly screen potentially new antimicrobials and to be as accurate, quick, and efficient as possible. Existing methods are still too destructive and often require the removal of the formed biofilm from the growth substrate to accurately evaluate the number of live bacteria. Indeed, for the MBEC Assay or the BRT, which is a static device, the limited nutrients supplied can only roughly mimic the physiological context of human infections.

**Figure 3.**
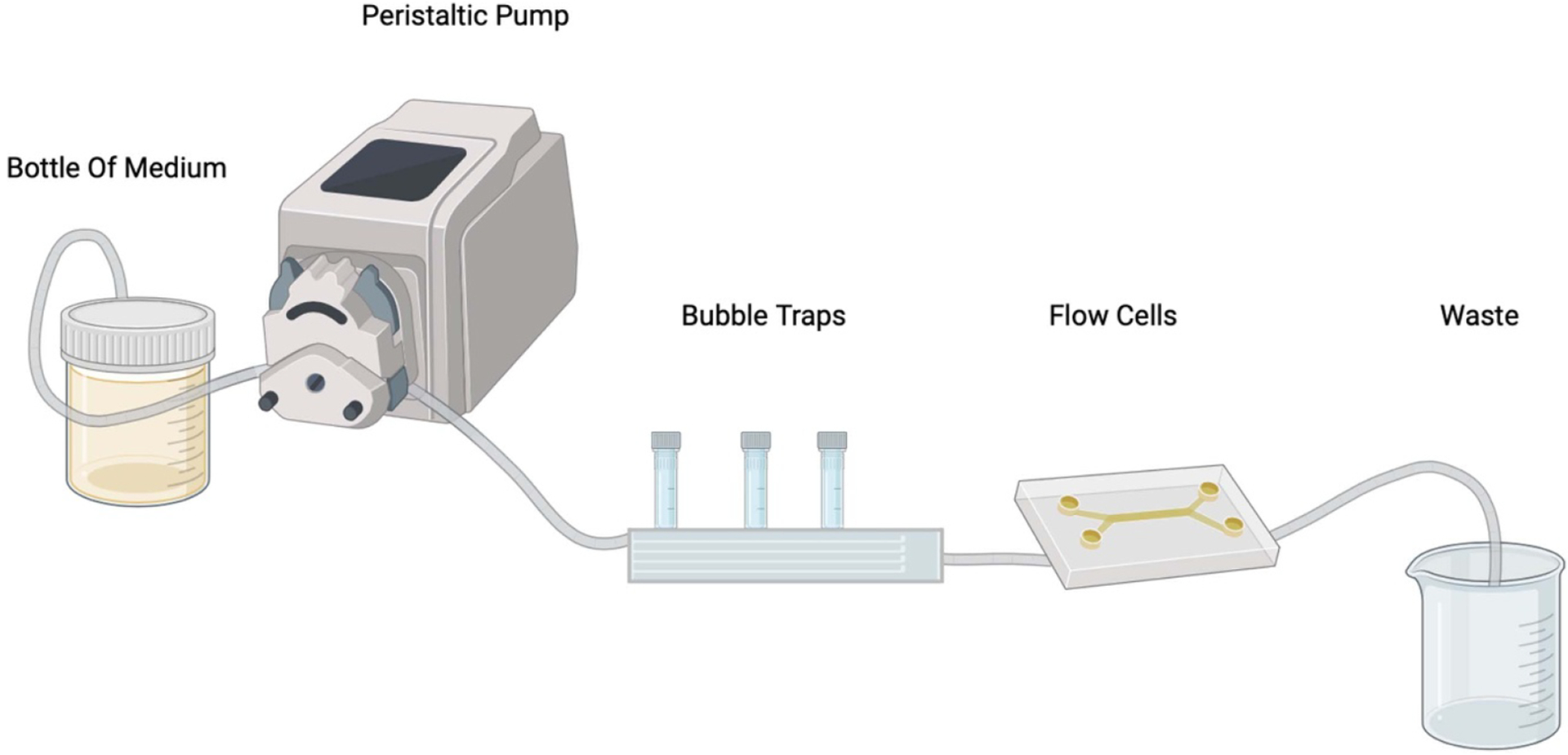
Flow cell model. Bacteria are inoculated in flow cell chamber, specifically designed for microscopy observation, where the biofilm develops. The flow is created thanks to the peristaltic pump and goes through the flow cells. This scheme created in BioRender.com.

An alternative model for studying biofilms is flow cells (figure 3). In this system, a peristaltic pump allows the flow of nutrients and the antimicrobial agent of interest, and the biofilm develops over time on the plastic chamber covered with a microscopy-compatible coverslip. This model has been very useful in the testing of antibiofilm compounds and was used to study the dispersal of cells from biofilms upon treatment with compounds (de la Fuente-Núñez *et al* 2014, Reffuveille *et al* 2014, Varin-Simon *et al* 2021). The need for microscopy to visualize the results obtained perhaps makes this approach less accessible. Other dynamic models, such as the Rotary reactor or Robbins device (Pacha-Olivenza *et al* 2019, Cantillon *et al* 2021), also have drawbacks, including the decrease of planktonic bacteria due to the flow or a decrease in communication between planktonic and adherent bacteria. Moreover, experiments conducted in a reactor require a large volume of media and numerous tubes (*e.g.,* for 6 samples, using two different flow-cells, would require 6 tubes of 1.2 m and 50 mL of culture medium per day, consuming a high quantity of molecules to screen) and can represent quite a massive installation with a unique condition output per assay; thus, these dynamic models may not be suitable for high-throughput screening efforts.

An alternative approach is represented by xCELLigence. This device makes use of electrical impedance spectroscopy based on a defined Cell Index (CI) parameter. The electrode records an electrochemical reaction and detects the changes in the diffusion coefficient of a redox solute. This technology has been integrated into a microfluidic chamber, allowing the bacteria to attach and the biofilm to grow, yielding a system called the BiofilmChip Blanco-Cabra *et al* 2021. This method (BiofilmChip), which uses impedance to measure cellular mass, is suitable for real-time and *in situ* measurements independent of confocal microscopy (Blanco-Cabra *et al* 2021).

Microfluidics and other new tools are also being developed. For example, Bioflux has made a system with a microfluidic chamber aligned to an interdigitated electrode where the biofilms grow. The addition of a prechamber prevents biofilm disturbances caused by manual inoculations that suddenly increase the flow rate (and consequently the shear stress) inside the chambers, which is one of the key points to consider when adapting microfluidic devices to studies of biofilm growth and other measurements (Naudin *et al* 2019).

### 3.2. Engineering approaches

Engineering approaches have emerged as an alternative to classical antimicrobial molecule development efforts. Synthetic peptides are being produced and screened to find those with the best antibiofilm activity. *In silico* analysis of the structure/activity relationships of molecules have enabled the design of peptides that are more efficient against biofilms (Haney and Hancock 2013, Haney *et al* 2018).

Antibiofilm compounds may also be functionalized onto surfaces to protect such surfaces from invading bacteria that are likely to form biofilms. Functionalization may involve grafting molecules onto the surface or creating textured surfaces. For example, polymer brushes can be grafted to achieve a bacteriostatic or bactericidal effect (Dhingra *et al* 2022), and amine-grafted inorganic adsorbents can be designed to remove bacterial pollutants from water (Bouazizi *et al* 2022). There is a wide variety of grafting methods; however, the attached molecule must be stable, effective when bound, and non-cytotoxic for medical applications. Textured surfaces can be used to obstruct bacterial colonization and biofilm development by limiting the adhesion of bacterial cells to the surface or by killing the bacteria owing to the ‘sharp’ nano-relief that disrupts bacterial membranes, without having to apply chemical treatments, which have associated toxicity. Many nanotexturing patterns, simple and complex, based on observations from nature, have been tested as antibiofilm methods. For example, Rostami *et al* (2021) tried to artificially reproduce shark skin, which is highly hydrophobic and prevents bacterial adhesion (Rostami *et al* 2021). Some insects possess nanometric roughness, which induces membrane disruption followed by bacterial lysis (Ivanova *et al* 2012, Pogodin *et al* 2013). However, to develop this nanotechnology for healthcare materials, issues related to cytotoxicity and poor tolerance by the human host still need to be addressed (Jaggessar *et al* 2017).

## 4. Future directions: the promise of computers for designing antibiofilm molecules

**Figure 4.**
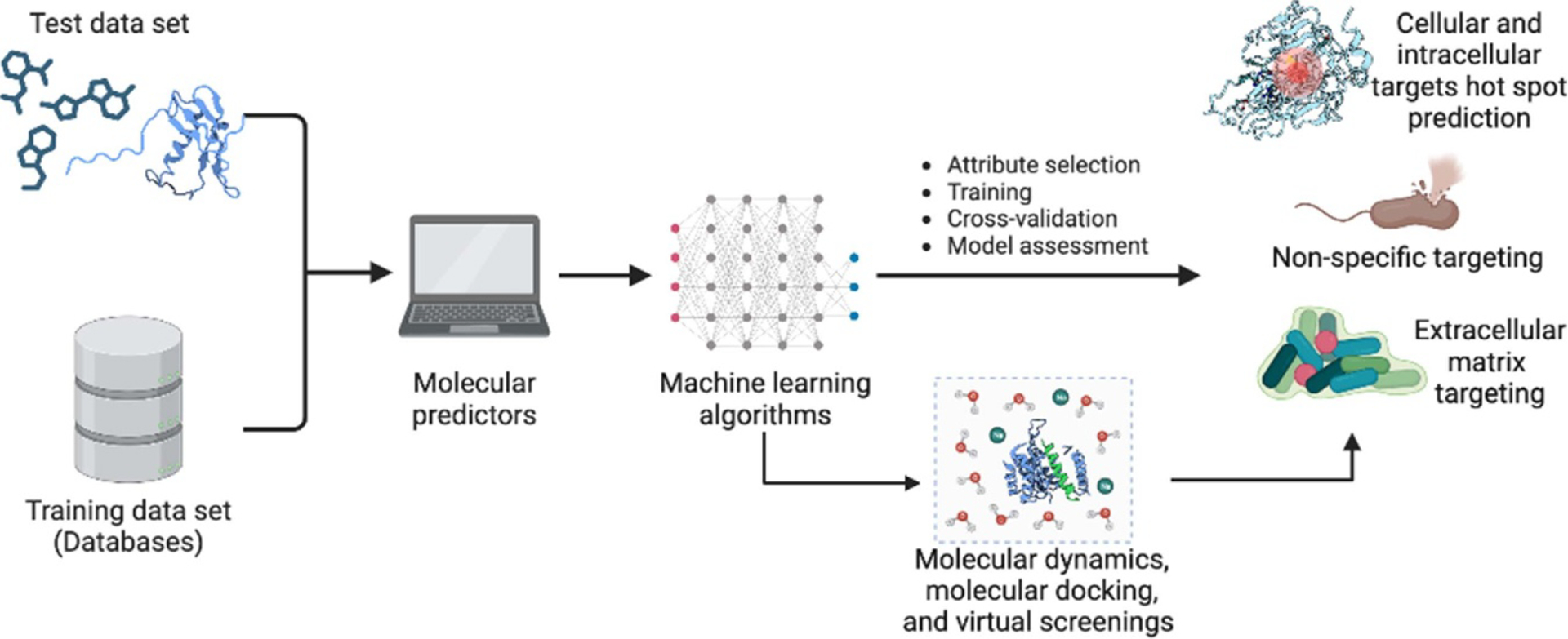
Computational methods for the prediction of antibiofilm compounds. Machine learning methods, coupled with molecular dynamics, molecular docking, and virtual screenings can be used to accelerate the discovery of antibiofilm compounds with specific or non-specific targets. This figure was created in BioRender.com.

Computational methods have become a valuable tool in the discovery of new antibiofilm molecules (Cardoso *et al* 2018, An *et al* 2021) (figure 4), opening the possibility to predict the efficacy of potential compounds, design more effective drugs (Torres *et al* 2022, Maasch *et al* 2023, Wong *et al* 2023), and simulate complex molecular interactions. Molecular docking was one the first methods employed for the study and prediction of antibiofilm compounds. It involves predicting the binding affinity of a molecule to a specific target protein with in the biofilm matrix, bacterial membrane, or an intracellular protein target. This process aided researchers in modeling the interaction between a potential drug and its target protein to predict the drug’s efficacy and optimize its chemical structure to enhance binding affinity. However, the computational cost of such studies and the restrictions in obtaining targets are a hurdle for the broad use of this technique. Alternatively, molecular dynamics simulations have also been used to provide insight into structural and dynamic features of antibiofilm compounds. Although these simulations yield a highly detailed set of features of antibiofilm compounds, this technique has been less explored for the generation of antibiofilm molecules because of the high complexity of the biofilm environment that counts with too many variables, such as the specific bacterial strains, biofilm architecture, *pH*, and extracellular polymeric substances, making it costly and less accurate with the approximations usually adopted in these simulations. Virtual screenings and quantitative structure-activity relationship (QSAR) modeling have been the most commonly used computational techniques over the years because of the low need for computational power and high accuracy in predicting antibiofilm compounds (de la Fuente-Núñez *et al* 2015a, Haney *et al* 2018, Agbale *et al* 2019). In virtual screenings, large databases with biologically active compounds, not necessarily against biofilms, are searched for similarities with known antibiofilm agents. On the other hand, QSAR modeling predicts the activity of a compound based on its physicochemical properties and structure.

While these methods have been useful in identifying potential hits, they are limited by their reliance on expert-designed features and lack of flexibility, and despite the hits found using these methods, the identification of effective antibiofilm compounds has been slower than the rate of appearance of resistant microorganisms. In this sense, machine learning (ML) represents a significant advance over traditional computational methods such as QSAR, virtual screening, and molecular docking to identify novel antibiofilm agents. Indeed, ML has gained significant attention in recent years due to its ability to learn and adapt to complex data and make predictions with high accuracy, even in the absence of predefined features. ML algorithms can analyze large amounts of data and identify patterns and relationships that are not easily discernible to humans or other computational techniques (Rahman *et al* 2022), such as QSAR, virtual screening and molecular docking.

Various ML models have been successfully applied to predict the antibiofilm activity of new compounds based on their chemical structures and other molecular features. For example, the ML-based virtual screening approach (Rahman *et al* 2022) was used to categorize 29 537 compounds based on their growth inhibitory activity (hit rate 0.87%) against the antibiotic-resistant bacterium *Burkholderia cenocepacia*. Next, a directed-message passing neural network was used to describe the molecular features of these compounds. Training an ML model on this data resulted in a receiver operating characteristic score of 0.823 on the test set. Subsequently, this model was applied to predict antibacterial activity in virtual libraries, including 1614 compounds from the Food and Drug Administration (FDA)-approved list and 224 205 natural products. The top-ranked predicted compounds were experimentally validated with higher hit rates of 26% and 12% for the FDA-approved and natural compounds, respectively. These rates represent at least a 14-fold increase compared to previous screening efforts. Furthermore, more than 51% of the predicted antibacterial natural compounds exhibited inhibitory activity against ESKAPE (*Enterococcus faecium, S. aureus, Klebsiella pneumoniae, Acinetobacter baumannii, P. aeruginosa,* and *Enterobacter* species) pathogens. Another example is TargIDe (Carneiro *et al* 2023), a ML-based consensus workflow that predicts the protein targets of molecules known to inhibit biofilm formation by *P. aeruginosa*. The workflow utilizes a specialized database containing all known targets involved in biofilm formation by this bacterium. The experimentally confirmed inhibitors available on ChEMBL, along with chemical descriptors, served as input features for a combination of nine classification models, including *k*-nearest neighbors, support vector machine (SVM) with four different kernels, random forest, naïve Bayes classifier, and gradient-boosting decision trees (XGBoost). First, the models were trained with the ten most relevant features, and afterwards with combinations of four, five, and six of these features using median values of information gain (IG), IG ratio, Gini decrease, and chi-square (*χ*^2^). Interestingly, the classifiers that performed better were those that used less features as input, and predictions were more accurate for the targets with a higher number of ligands in the training set. The combination of these features, along with their complex relationship, would not be easily extracted without these ML models. This approach provides a consensus method to predict the most probable target of a ligand. Molib (Srivastava *et al* 2020), an approach based on ML-based classification models to predict the biofilm inhibitory activity of small molecules, is also a successful example of ML models used for the identification of antibiofilm compounds. For this model’s development, a training dataset of biofilm inhibitory molecules and selected structural and chemical features was curated, and through algorithm optimization, the model yielded high accuracies of 0.93, 0.88, and 0.90 for Random Forest-based descriptor, fingerprint, and hybrid classification models, respectively. The performance of these models was further evaluated on separate validation datasets containing biofilm inhibitory and non-inhibitory molecules, consistently achieving accuracy levels of ⩾0.90. The Molib web server represents a reliable and highly useful tool for predicting the biofilm inhibitory activity of small molecules.

There are also instances where ML models can be combined with QSAR or molecular docking and yield much faster and optimized virtual screenings. For instance, the combination of an ML model, a pharmacophore based virtual screening, molecular dynamics simulations, and molecular docking (Vetrivel *et al* 2023) to search for antibiofilm inhibitors with a specific target in *P. aeruginosa* (LasR gene), which plays a key role inquorum sensing during biofilm formation. This multi-component model used a SVM learning algorithm to generate QSAR models based on a dataset of 66 antagonists. The top three models achieved correlation coefficients (*R*^2^) of 0.67, 0.86, and 0.91. Next, a four-point pharmacophore model (AAAD_1) was developed and employed to screen the MolPort database using ZincPharmer. Compounds that exhibited predicted pIC_50_ values >8, as determined by the SVM models, underwent docking analysis to rank them based on docking scores. Four top leads, namely ZINC3851967, ZINC4024175, ZINC2125703, and ZINC3851966, were selected. Molecular dynamics simulations were conducted for 100 ns to assess the stability of these compounds, and their absorption, distribution, metabolism, and excretion properties were validated, resulting in promising antibiofilm compounds with specific mechanism of action.

## 5. Conclusion

Advances in the past decade have advanced our understanding of biofilms and antibiofilm approaches, opening the door for developing therapies that specifically target biofilms. We anticipate that the use of artificial intelligence will help accelerate our ability to discover much-needed molecules to counter infectious biofilms.

## Figures and Tables

**Table 1.** Comparison between different antibiofilm approaches.

Antibiofilm approach	Advantages	Disadvantages
Antibiotics	Specific bacterial targets Efficiency against biofilm up to 100% for prevention but rarely more than 90% for eradication	Small colony variants formation Inefficient against resistant strains Antagonism
Bacteriophages	Additional to antibiotics Total eradication on proximal femoral	Need clinical trials
Probiotics	Total removal of pathogenic biofilm	Still a biofilm, even if non-pathogenic one Few probiotic strains efficient
AMPs	Low concentration (<20 *μ*g ml^−1^) Synergy with antibiotics Broad spectrum Peptides can be designed with specific anti-biofilm activity Complete inhibition and eradication of biofilm	Need clinical trials

## Data Availability

No new data were created or analyzed in this study.
